# Defects of Hearing

**Published:** 1916-05-13

**Authors:** 


### THE SCHOOL DOCTOR.

### Defects of Hearing.

Of all physical hindrances to education, defective hearing is perhaps the best known and yet very frequently overlooked. The most experienced medical officer is startled at times to realise the extent to which very serious deafness may go undetected for years, in spite of trained teachers and periodic medical examinations. The totally deaf are usually noted and segregated, but partial deafness still receives very inadequate recognition, and is responsible for much educational waste. A clever child teaches itself a certain amount of lip-reading, and by watching the teacher's face and gestures and copying the movements of other children, it may be able to keep up with the work of the class to a surprising extent. Duller deaf children are often suspected of mental deficiency or wilful inattention; undeserved punishment, combined with a constant effort to catch indistinct sounds, may set up a serious condition of nerve strain which brings them under the notice of the school doctor. Bad acoustics in the classroom, noisy school surroundings, and a bad articulation or shrill, high-pitched voice in the teacher greatly aggravate the miseries of partially deaf children, who are more numerous than is commonly supposed, though accurate estimates are wanting.

In few conditions is early diagnosis more important; whatever the cause of deafness, the prognosis of success in medical and educational treatment depends directly on the time at which the condition is brought under treatment. Yet the detection of hearing defects presents many unexpected difficulties. The theoretically ideal method of testing every individual child at routine medical inspections may be rejected at once as impracticable. Formal hearing tests depend enormously on intelligence in the child and quiet in the surroundings, and even if the school doctor had unlimited time in which to overcome stupidity and shyness, the horribly noisy conditions prevailing in the vast majority of schools would entirely vitiate his results. A much more effective plan is to pick out, with the help of the teachers, suspected cases of bad articulation, marked inattention, mental retardation, adenoids, and otorrhœa, for special testing at the end of the school session, when reasonable quiet can be secured. Teachers can render much assistance by rearranging the class for dictation or mental arithmetic so that every child has a turn in the back row.

### Otorrhœa.

The question of the medical treatment of deafness, in and out of school, centres almost entirely on the subject of discharging ears. It is unfortunate that this disease should be officially dubbed a "minor ailment," for it is one of the most important of child life. Middle-ear disease is responsible yearly for 600-700 deaths in children under fifteen, and about half the unfortunate children in deaf schools are there from this cause. It is responsible for a still larger proportion of partial deafness in childhood and for a considerable amount of the defects of hearing which appear in later life. An interesting report by a school medical inspector in the West Riding of Yorkshire in 1915 shows that in one area children with ear discharge were nine times as likely to die during school life as children with healthy ears.

Of the known causes of middle-ear disease, the most important, neglected naso-pharyngeal obstruction, lies within the school doctor's sphere of influence, and it is in connection with the prevention of deafness that he can most strongly urge on his authority the necessity for providing adequate treatment for tonsils and adenoids. A large percentage of the cases are attributed to the acute exanthemata, measles being the chief culprit with about 8 per cent. Prevention here belongs to another branch of public health administration, but it may be anticipated that the notification of measles, if accompanied by the provision of nursing care, will lead before long to an appreciable diminution in the cases of otorrhœa and deafness seen in school.

### The Importance of Treatment.

Once ear discharge has been established the serious difficulties of the school doctor begin. The minimum medical needs of these cases have been established beyond all discussion by the experience of well-organised hospital aural departments and of school clinics, such as Dunfermline. The necessary routine includes a thorough preliminary examination by a practitioner of experience, followed by re-examination at short intervals, prompt removal of any septic focus in pharynx, nose, or mouth, daily *skilled* nursing treatment, and a medical operation in intractable cases. On none of these points is successful compromise or cheese-paring possible. The hospital which offers examination by an aural specialist, and then hands the mother a packet of boracic powder and a bottle of drops, and instructions to return in a fortnight, is largely responsible for the popular notion that running ears are a dispensation of Providence with which it is useless or even dangerous to interfere. Nor does the opposite policy, adopted by some education authorities, of relying on rule-of-thumb nursing treatment, with only perfunctory or inexpert medical supervision, prove more of a success. The fact that treatment must be carried out daily, or in bad cases twice daily, makes the question of accessibility very important to working-class parents, and would even justify the use of the school premises in rural districts. The quite probable alternatives of deafness or early death are grave enough to excuse serious breaches with tradition, especially when experience has proved that, given proper treatment, practically every case is curable. Voluntary hospitals are doing much, but for the most part the solution must lie in extension of school clinics.

The educational treatment of the deaf is a problem, first, of early recognition, and then of careful classification. The former is important whether the defect is congenital or acquired, for the congenital cases are irreparably damaged in intelligence if their special education is long delayed, and the older children lose their speech with astonishing rapidity. Efforts are being made to substitute three years for seven as the age at which authorities are legally empowered to provide training. In classifying the children, it is usually found that those who cannot hear a forced whisper at one metre must be educated as "deaf"; those who can hear beyond two metres can make sufficient progress if kept in the front class of an ordinary school. The intermediate cases, who pick up sounds between one and two metres, should be trained in "hard-of-hearing" classes. The guiding principle throughout should be to keep the child in the highest class of school in which she can make reasonable progress, so essential is it to prevent the development of the peculiar mentality and habits of the deaf, which unfit the victim more and more for normal life. The "hard-of-hearing" classes should therefore be attached to an ordinary, and not a deaf, school, and the "partially deaf" children should mix with their normal fellows in play, manual classes, and physical exercises, separate instruction in speech and lip-reading being provided. The co-operation of the parent must be enlisted to prevent the growth of a gesture language. Some of the children may in time be returned to the ordinary school, but in all the remarkable improvement in brightness and happiness and intellectual attainments is one of the most gratifying features of the specialist side of school hygiene.

